# The role of general practitioners in dementia diagnosis: a scoping review of clinical practice guidelines

**DOI:** 10.1093/fampra/cmaf103

**Published:** 2026-01-22

**Authors:** Mary Cronin, Aisling Jennings, Nicola Cornally, Irene Hartigan, Séan O'Dowd, Marieke Perry, Suzanne Timmons, Kieran Walsh, Tony Foley

**Affiliations:** Department of General Practice, School of Medicine, College of Medicine and Health, University College Cork, Cork, T12 XF62, Ireland; Department of General Practice, School of Medicine, College of Medicine and Health, University College Cork, Cork, T12 XF62, Ireland; School of Nursing and Midwifery, University College Cork, Cork, T12 AK54, Ireland; School of Nursing and Midwifery, University College Cork, Cork, T12 AK54, Ireland; HSE National Dementia Services, Central Business Park, Clonminch, Tullamore, R35 F6F8, Ireland; Department of Geriatrics, Radboud University Medical Centre, 6500 HB Nijmegen, The Netherlands; Centre for Gerontology and Rehabilitation, School of Medicine, College of Medicine and Health, University College Cork, Cork, Ireland; School of Pharmacy, University College Cork, Cork, Ireland; Department of General Practice, School of Medicine, College of Medicine and Health, University College Cork, Cork, T12 XF62, Ireland

**Keywords:** dementia, general practitioners, clinical practice guidelines, diagnosis, general practice, primary care, scoping review, disease-modifying therapies, biomarkers, cognitive testing

## Abstract

**Background:**

Timely diagnosis of dementia is a public health priority, with general practitioners (GPs) central to symptom recognition and assessment. The emergence of biomarkers and anti-amyloid therapies makes accurate, timely diagnosis more critical than ever, introducing new complexities for general practice. Clinical practice guidelines (CPGs) are vital tools to support clinical decision-making, but their applicability to the general practice setting is uncertain.

**Objectives:**

This scoping review analyses how international CPGs define and support the GP's role in the dementia diagnostic process.

**Methods:**

Following the Arksey and O’Malley scoping review framework, five electronic databases and multiple grey literature sources were searched for dementia CPGs published between 2019 and 2025. Guideline quality was assessed using selected domains of the Appraisal of Guidelines for Research & Evaluation II instrument (AGREE II).

**Results:**

Fifteen CPGs from a range of healthcare systems were included. Only two were specifically developed for general practice. While most CPGs positioned GPs as key to timely diagnosis, the recommendations were predominantly developed from a secondary-care perspective and failed to address the fundamental barrier of limited consultation time. Furthermore, practical guidance for GPs on integrating new biomarkers and anti-amyloid therapies was almost absent.

**Conclusions:**

A disconnect exists between CPG recommendations and the realities of general practice, rendering much of the guidance aspirational rather than actionable. To be effective, future guidelines must ensure recommendations are feasible, address resource constraints, and establish clear pathways for the new biological era of dementia care. Without this, general practice will remain ill-equipped to meet the growing challenges of dementia diagnosis and management.

Key messages

- CPGs often focus on secondary care, overlooking general practice contexts.
- CPGs overlook GPs’ workload, making advice aspirational, not actionable.
- CPGs lack guidance on integrating new biomarkers and anti-amyloid therapies.
- Future guidelines must be adapted to the realities of general practice.

## Introduction

Dementia care is a global health priority, acknowledged as one of the most significant challenges in health and social care today [1]. Globally, dementia ranks as the seventh leading cause of mortality [2], with over 50 million people living with dementia worldwide, a number that is projected to increase to 153 million by 2050 [3, 4]. A timely diagnosis can significantly improve the quality of life of people with dementia by facilitating symptom management, implementing coordinated care plans, modifying risk factors, enabling access to appropriate support services, and affording the ability to participate in discussions and decision-making about future care [5–7]. Furthermore, the recent approval of the first anti-amyloid therapies (AATs) and the emergence of biomarkers are fundamentally changing the diagnostic approach for Alzheimer's disease, shifting the focus from a purely clinical diagnosis to one supported by biological evidence. This makes a timely and accurate diagnosis more critical than ever. A timely diagnosis is the gateway for assessing patient suitability for these novel treatments, at a disease stage where they are more likely to be eligible according to current licensing, i.e. mild cognitive impairment and mild dementia only [8]. However, these therapies are not a cure. They offer modest benefits, slowing the rate of cognitive decline by approximately 27% over 18 months in a key clinical trial [9]. This must be balanced against significant clinical considerations. A key risk is amyloid-related imaging abnormalities (ARIA), which can present as swelling or bleeding in the brain. While often asymptomatic, ARIA requires careful monitoring and can sometimes cause clinical symptoms. These treatments also come with heavy logistical burdens, including frequent infusions and monitoring, which pose substantial challenges for healthcare systems and will introduce significant new demands on general practice for complex patient counselling, timely referral, and care coordination.

Despite compelling evidence of the benefits of timely diagnosis and intervention, dementia remains widely underdiagnosed [10–12]. For example, a cross-sectional observational study in the USA found that only 41% of older adults with probable dementia were both diagnosed and aware of their diagnosis [13]. Barriers to diagnosis in general practice have been summarised across three main categories: patient factors, often caused by consciously or unconsciously delayed presentation; general practitioner (GP) factors, including diagnostic uncertainty; and system factors, such as significant time constraints inherent in general practice consultations, complex referral and diagnostic pathways, and a lack of post-diagnostic support [14, 15].

As the usual first point of contact for patients, GPs play a pivotal role in symptom recognition, initial assessment, clinical reasoning, and onward referral for confirmation of diagnosis and sub-typing, as well as providing continuing care coordination and support. While GPs have faced criticism for low rates of dementia diagnosis in primary care [16–18], research also indicates that many GPs adopt a nuanced, patient-centred approach [19]. This involves weighing up the risks and benefits of making a diagnosis of dementia [20], often activating various dementia care services and support systems for their patients, even in the absence of a formal diagnosis [19]. GPs carefully consider the moment to start the investigation and the diagnostic process that will minimize harm to patients and their relatives.

Given the complexities in dementia diagnosis and management, clear and practical guidance for GPs is paramount. Clinical practice guidelines (CPGs) are designed to assist healthcare practitioners in making informed decisions about patient care based on the best available evidence [21]. While previous reviews of CPGs have examined aspects of dementia care, including Alzheimer's disease [22], dementia risk reduction [23], the management of behavioural and psychological symptoms [24] and the diagnosis and management of mild cognitive impairment [25], a rigorous analysis of the recommendations regarding the specific role of GPs in the diagnostic process of dementia has not been previously undertaken.

This scoping review explores how CPGs define and support the GP's role in dementia diagnosis.

Key objectives are as follows:

- To map the recommendations regarding the role of GPs in the dementia diagnostic and referral pathway.
- To explore how CPGs address key aspects of dementia diagnosis in general practice, including symptom recognition, clinical assessment, investigation, and diagnosis.
- To identify recommendations regarding the introduction of biomarkers in the diagnostic process and novel AATs for Alzheimer‘s disease.
- To identify gaps and variations in guideline recommendations related to GPs’ responsibilities in the diagnosis of dementia.

## Methods

This scoping review adhered to the methodology outlined by Arksey and O'Malley [26–28] with reporting guided by the Preferred Reporting Items for Systematic Reviews and Meta-Analysis extension for scoping reviews (PRISMA-ScR) [29]. The quality of the included CPGs was assessed using the Appraisal of Guidelines for REsearch & Evaluation II (AGREE II) instrument [30]. This scoping review protocol was registered with Open Science Framework on 17th June 2024 and published on the Health Research Board (HRB) open research website in July 2024 [31].

### Search strategy and identifying relevant papers

The search strategy was developed by the first and last authors, refined by the research team, and reviewed by a librarian from University College Cork. Search terms were adapted for each database; the full strategy is provided in Supplemental File S1. Initial searches were carried out between July and August 2024. The comprehensive search was updated in February 2025, before final analysis, to ensure the inclusion of the most up-to-date evidence.

We included CPGs published within the last 5 years to ensure clinical currency. While our primary focus was on evidence-based CPGs, we also included consensus statements where their recommendations were deemed sufficient to inform practice and other inclusion criteria were met. No language restrictions were applied; non-English CPGs were translated using Google Translate [32]. We excluded CPGs for specific populations (e.g. intellectual disability, early-onset dementia) and those specific to non-primary care settings or targeting other healthcare professionals (e.g. nurses, allied healthcare professionals). A complete list of inclusion and exclusion criteria is in Supplemental File S1.

We searched five peer-reviewed databases: PubMed, CINAHL, Embase, PsycINFO, and Cochrane Library. An alert system was maintained on these databases until February 2025 for ongoing updates, but no records were included beyond February 2025. Recognising that CPGs may not be published in peer-reviewed journals, we conducted extensive grey literature searches, including Lenus (the Irish Health Repository), National Institute for Health and Care Excellence (NICE) and Scottish Intercollegiate Guideline Network (SIGN) websites and generic search engines (Google and Google Scholar, reviewing the first 200 citations). We also searched guideline databases (e.g. Guideline Central, Guidelines International Network (GIN), ECRI, TRIP, and the Agency for Healthcare Research and Quality) using adapted combinations of the bibliographic database search terms. Websites of national and international GP professional organisations were additionally searched. Where guidelines were unavailable or access restricted, we emailed organisations to request publications since 2019, using Google Translate for international correspondence. Reference lists of retrieved CPGs were also hand-searched. Citations were uploaded to Covidence software for data management and de-duplication. Title and abstract screening, along with full-text review, were completed independently by MC and TF. Any disagreements were resolved by consulting a third author (AJ).

### Charting and data synthesis

The data extraction tool was iteratively developed and piloted by MC and AJ. This involved independent extraction from three CPGs, followed by consultations to refine the tool. A further three CPGs were then extracted to finalize the tool's structure. Data extraction was conducted by one author and independently verified by another. Extracted data encompassed recommendations on the GP's role in dementia diagnosis, investigations, communication of the diagnosis, and referral.

### Quality assessment

The quality of the included CPGs and consensus statements was assessed using the AGREE II instrument [33]. The purpose of the AGREE II is primarily to assess the quality of CPGs [30]; however, it is commonly used to appraise consensus statements despite differences in the development process [34, 35]. As the focus of our study was on the diagnostic process and given the differences between CPGs and consensus statements, we determined that the full application of the AGREE II may not be an equitable platform for comparison. For this reason, it was determined that AGREE II was best applied by evaluating the guidelines using domains 1 (Scope and Purpose), 3 (Rigour of Development), and 4 (Clarity of Presentation) [35–38]. Two researchers (MC and AJ) independently scored each of the CPGs. For the Irish guideline, to which AJ contributed, it was pre-agreed that a third appraiser would be consulted if consensus could not be reached. Differences greater than two points for any item were resolved through consultation and discussion. Domain scores were calculated using the AGREE-II formula [30].

## Results

### Included guidelines

**Figure 1:**
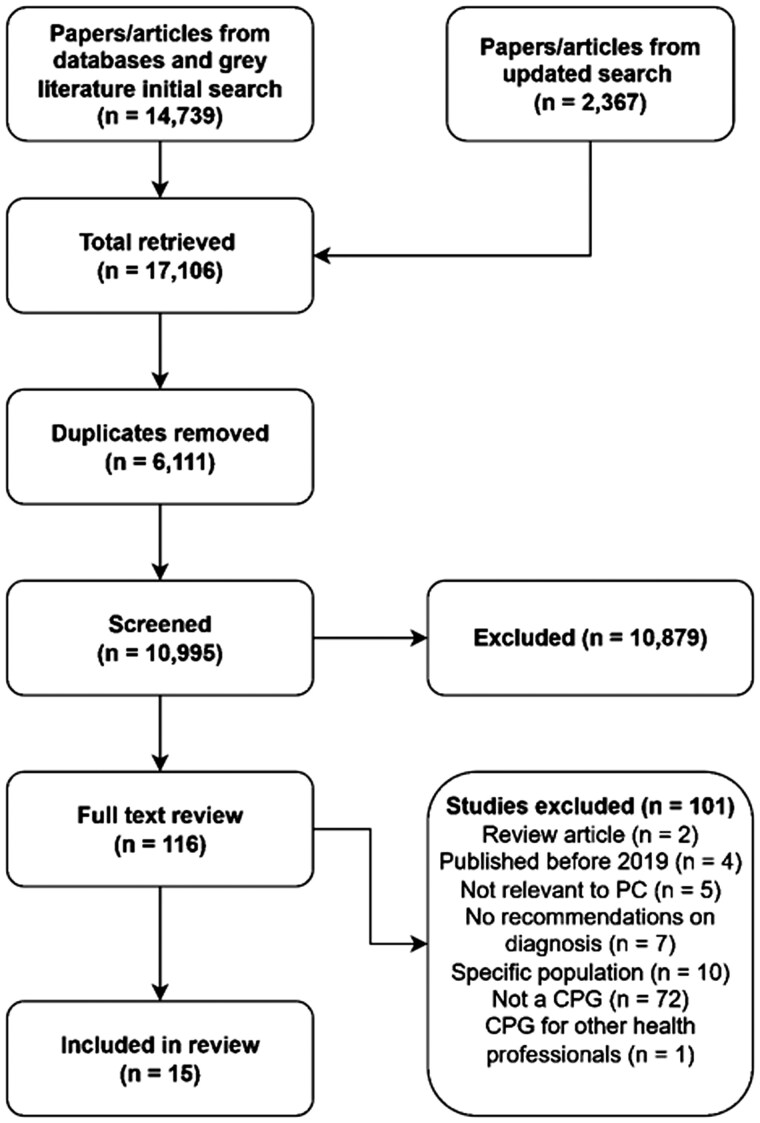
PRISMA diagram. This figure illustrates the identification, screening, full-text review, and inclusion of studies in the review following PRISMA Extension for Scoping Reviews (PRISMA-ScR) guidelines.

A total of fifteen documents met the inclusion criteria (Fig. 1). Of these, eleven were classified as CPGs, originating from Turkey, Finland, Qatar, the Netherlands, Norway, Germany, Ireland, Malaysia, Scotland, Italy, and the USA. The remaining four, originating from Canada, Argentina, Latin America, and Brazil, were identified as consensus statements or recommendations. All originated from countries classified as high or upper-middle-income by the World Bank [39]. For reporting consistency, all included documents are hereafter referred to as CPGs. Table 1 lists the included CPGs by publication year.

**Table 1:** Characteristics of included studies.

Country of origin	Authors/Institution	Title	Publication year	Target healthcare audience
USA[40 ]	Atri et al/Alzheimer's Association	DETeCD-ADRD: The Alzheimer's Association Clinical Practice Guideline for the Diagnostic Evaluation, Testing, Counselling and Disclosure of Suspected Alzheimer's Disease and Related Disorders: Comprehensive Report	2024	All clinicians, including those in primary, speciality (e.g. geriatrics, psychiatry, neurology, neuropsychology) or subspecialty care (i.e. dementia subspecialist)
Italy[41]	Fabrizi et al/National Institute of Health (requested by Ministry of Health)	Diagnosis and treatment of dementia and Mild Cognitive Impairment	2024	All health and social care professionals involved in caring for people with dementia or MCI in any setting
Norway[42]	Directorate of Health	Dementia National Professional Guideline	2024—update to 2017 version	Healthcare service providers responsible for investigating, diagnosing, treating, and following up with people with dementia.
Finland[43]	The Finnish Medical Society Duodecim, The Finnish Geriatric Association.	Memory Disorders—Current Care Guidelines	2023	Doctors and other health, social work, pharmacy professionalsand students in the field.
Germany[44]	DGN e. V. & DGPPN e. v. (Ed.)/German Society for Psychiatry and Psychotherapy, Psychosomatics and Neurology (DGPPN), German Society of Neurology (DGN)	S3 guideline Dementia Long Version	2023	Doctors from specialist departments of the participating professional associations, neuropsychologists, occupational therapists, physiotherapists, art therapists, music and dance therapists, Speech therapists, Nursing staff and social workers.
Latin America^a,b^ [45]	Lopera et al./Americas Health Foundation	A task force for the diagnosis and treatment of people with Alzheimer's disease in Latin America	2023	Health professionals
Scotland[46]	Scottish Intercollegiate Guidelines Network (SIGN)	SIGN168—Assessment, diagnosis, care and support for people with dementia and their carers	2023	Primary and secondary healthcare professionals, social care professionals, community, care home and care-at-home staff.
Brazil^a,b^ [47]	Jerusa Smid et al./Scientific Department of Cognitive Neurology and Aging of the Brazilian Academy of Neurology	Subjective cognitive decline, mild cognitive impairment and dementia-syndromic diagnosis recommendations	2022	Not Reported
Malaysia [48]	Ministry of Health Malaysia	Management of Dementia—Third Edition	2021	Primary, secondary and tertiary care settings
Canada^a^ [49]	Ismail et al./Canadian Consensus Conference	Recommendations of the 5th Canadian Consensus Conference on the diagnosis and treatment of dementia	2020	Clinicians, researchers, policymakers the lay public
Netherlands[50]	Dieleman-Bij de Vaate AJM, Eizenga WH, Lunter-Driever PGM, Moll van Charante EP, Perry M, Schep-Akkerman A, Smit BSJ, Starmans R, Verlaan-Snieders MNE, Van der Weele GM/Dutch College of General Practitioners (NHG)	NHG Standard Dementia (M21)	2020	General Practitioners
Qatar[51]	Ministry of Public Health Qatar	National Clinical Guidelines: The diagnosis & treatment of dementia.	2020	Healthcare professionals in primary care and outpatient settings
Turkey^b^ [52]	Turkey Ministry of Health General Directorate of Health Services	Alzheimer's and Other Dementia Diseases Clinical Protocol (Version 1.0)	2020	Physicians interested in the diagnosis and treatment of diseases that cause dementia, especially Alzheimer's disease.
Argentina^a,b^ [53]	Demey et al. /Buenos Aires Neurosciences program (Ministry of Health).	Recommendations for the detection and diagnosis of Dementia due to Alzheimer's disease in the city of Buenos Aires	2019	Health professionals at each level of care, from primary care to specialist care.
Ireland[54]	Foley et al./Irish College of General Practitioners (ICGP) Quality and Safety in Practice Committee	Dementia: Diagnosis & Management in General Practice	2019	General Practitioners

^a^Consensus Statement. ^b^No representation from general practice in development.

Of the fifteen included documents, only those from Ireland and the Netherlands were explicitly developed for the general practice setting [50, 54]. The remaining CPGs targeted a broader audience of health professionals. Notably, four CPGs did not report general practice representation in their development process, despite stakeholder representation from other health care professionals [45, 47, 52, 53].

### Quality appraisal

Assessment using the AGREE II tool revealed varied quality across the included CPGs (Table 2). All CPGs scored highly (>80%) in Domain 1 (Scope and Purpose), indicating clear objectives and target populations. In Domain 3 (Rigour of Development), 10 of 15 CPGs scored >70%, demonstrating transparency in systematic methods, evidence selection, and external review. While the consensus statements from Canada (84%) and Argentina (75%) scored highly in Domain 3, the consensus statement from Brazil scored poorly (14%), as did the CPGs from Turkey (27%) and Finland (46%). In Domain 4, nearly all CPGs, except for Brazil (47%) and Turkey (53%), scored >70% (Clarity of Presentation), signifying clear and accessible language.

**Table 2:** AGREE II domain scores.

Country of origin of the guideline	Scope and purpose Domain 1 (%)	Rigour of development Domain 3 (%)	Clarity of presentation Domain 4 (%)
**Scotland (SIGN)**	97	99	89
**Malaysia**	100	95	94
**Canada** ^a^	100	84	94
**Germany**	97	79	94
**Netherlands**	97	75	100
**Argentina** ^a^	94	74	92
**Brazil** ^a^	86	14	47
**Norway**	94	88	97
**Turkey**	94	27	53
**Finland**	69	46	74
**Qatar**	100	92	94
**Latin America** ^a^	97	45	78
**Ireland**	92	69	97
**Italy**	100	99	100
**America**	94	95	100

^a^Consensus Statement.

### Diagnostic Process

The CPGs uniformly addressed key aspects of the dementia diagnostic process, including clinical history taking, physical examination, blood tests, imaging, referral, and communication of the diagnosis. While all CPGs acknowledged the GP's central role in detection and timely symptom identification, the specific responsibilities varied significantly, as detailed in Table 3. All CPGs emphasized the importance of a detailed clinical history, with eight explicitly recommending that this should occur in the primary care setting [41, 45, 49–51, 53–55]. Key areas for assessment included decline in activities of daily living, cognitive function (e.g. memory impairment), changes in family and social relationships, sensory deficits (e.g. auditory or visual impairments) [41, 43, 45, 49–52, 55] and potential psychiatric symptoms, like mood disturbances [43, 55].

**Table 3:** Summary of the diagnostic process and the role of the GP.

	Argentina	Canada	Scotland	Malaysia	Latin America	Brazil	Ireland	Germany	Norway	Netherlands	Qatar	Finland	Turkey	Italy	USA
Clinical History															
Is the content of clinical history outlined?	☑	☑	☑	☑	☑	☑	☑			☑	☑	☑	☑	☑	☑
Is the role of the GP explicitly mentioned?	☑	☑			☑		☑			☑	☑			☑	☑
Is the involvement of an informant (relative) recommended?	☑	☑	☑	☑	☑	☑	☑	☑	☑	☑	☑	☑	☑	☑	☑
Physical examination															
Is physical examination outlined?	☑	☑	☑	☑	☑	☑	☑		☑	☑	☑	☑	☑	☑	
Is the role of the GP clearly stated?							☑		☑	☑	☑	☑		☑	
Cognitive assessments															
Is the role of the GP in cognitive assessments clearly stated?	☑	☑	☑		☑	☑	☑	☑	☑	☑	☑			☑	☑
Are specific assessments for use by GP outlined?					☑		☑	☑		☑				☑	☑
Investigations (Blood work)															
Are recommended blood tests outlined?	☑			☑	☑	☑	☑	☑	☑	☑	☑	☑	☑	☑	☑
Is the role of the GP in blood work specified?	☑						☑		☑	☑	☑				
Investigations (Imaging)															
Are imaging recommendations made?	☑	☑	☑	☑	☑	☑	☑	☑	☑		☑	☑	☑	☑	☑
Is the role of the GP in imaging specified?	☑						☑		☑		☑				
Disclosure of diagnosis															
Is guidance given on the disclosure of the diagnosis?			☑				☑		☑	☑	☑			☑	☑
Is the role of the GP in disclosure specified?							☑		☑	☑					

The importance of obtaining a collateral history from family members or caregivers was widely highlighted for accurate symptom reporting, given patients’ potential limited insight. However, procedures for this crucial information gathering were often ill-defined. Three CPGs suggested using questionnaires to assist collateral history-taking [46, 48, 49], while another three specified that the collateral history-taking should be conducted separately from the patient interview [50, 51, 53]. Five CPGs also included a physical exam as part of the GP's diagnostic role [42, 50–52, 54]. The CPGs offered limited guidance on the practical management of the overall assessment process in general practice. The Norway CPG suggests the GP can delegate part of the assessment, such as assessment of functional ability, to a wider multidisciplinary team who report back to the GP [42]. The SIGN guideline indicates that some assessments can be done by Allied Health Professionals [46], and the Qatar CPG suggests that an occupational therapist can complete the activities of daily living assessment [51].

### Cognitive tests

All included CPGs recommended cognitive testing, with the GP's role in this process evident in twelve [41, 42, 44–47, 49–51, 53–55] (see Table 3). Of these, CPGs from Latin America, Ireland, Germany, Italy, USA, and the Netherlands explicitly outlined specific tests appropriate for primary care [41, 44, 45, 50, 54, 55]. These include the Mini-Mental State Examination (MMSE), Montreal Cognitive Assessment (MoCA), and Mini-Cog. Table 4 summarises the cognitive tests recommended for use in primary care.

**Table 4:** Cognitive tests recommended for primary care.

CPG	Cognitive test recommended
Latin America	MoCA, MMSE, RUDAS, BCSB
Ireland^a^	GPCOG, Mini Cog, MIS
Netherlands^a^	MMSE, Clock drawing test, RUDAS
Germany	MMST, MOCA
Italy	10-CS, 6 CIT, 6 IS, MIS, Mini Cog
USA	MMSE, Mini Cog, MOCA, GPCOG

MoCA, Montreal Cognitive Assessment Test; MMSE/TMini-Mental State Examination/Test, RUDAS, Rowland Universal Dementia Assessment Scale; BCBS, Brief Cognitive Screening Battery; GPCOG, General Practitioner Assessment of Cognition; MIS, Memory Impairment Screen; 10-CS, 10 Point Cognitive Screener; 6-CIT, 6 Item Cognitive Impairment Test; 6-IS, 6 item screener. ^a^CGP was developed for the general practice/primary care setting.

### Diagnostic Criteria

CPGs frequently referenced established diagnostic criteria, predominantly from the Diagnostic and Statistical Manual of Mental Disorders (DSM-5), the International Classification of Diseases (ICD-10/ICD-11), or the National Institute on Aging and the Alzheimer's Association (NIA-AA). Eleven CPGs referenced DSM-5's clinical criteria [40, 41, 45–53], while six referred to ICD's clinical criteria [42, 44–48]. A biological approach to diagnosis, outlined by the NIA-AA criteria, was referenced in five CPGs [40, 41, 47, 49, 50]. Notably, these five CPGs also included one or more clinical classifications, often discussing the importance of clinical presentation being supplemented by biological markers.

### Investigations

Initial investigations when dementia was suspected, including blood tests and imaging, were outlined across the included CPGs. Five CPGs listed specific blood tests that were recommended as part of the diagnostic work-up [42, 50, 51, 53, 54]. However, blood tests were not explicitly part of the Scottish SIGN or Canadian CPG diagnostic process. The Netherlands CPG outlined blood tests that can be useful in cases of comorbidity, but suggested they have limited value in diagnosing dementia.

Recommendations for imaging varied significantly. CPGs from the Netherlands, Ireland, and Latin America designated imaging as a secondary care responsibility [45, 50, 54]. Conversely, the Qatar CPG recommended that brain magnetic resonance imaging (MRI) and computed tomography (CT) be requested in primary care. Similarly, the Norway, Argentina, and USA CPGs also recommended primary care ordering of imaging, with MRI often preferred [42, 53, 55].

Beyond specific diagnostic steps, some CPGs highlighted broader organizational and ethical considerations. The Scottish SIGN CPG emphasized that “primary care providers (GPs) should be made aware of person-centred approaches to care” and suggested that dementia care managers could benefit primary care [46]. The Latin America CPG voiced concerns that “opportunities for diagnosis may be missed because primary care providers (PCPs) often fail to screen older adults for AD due to insufficient time and training” [45]. In contrast, the Norway CPG outlined secondary care's role as complementing rather than replacing the GP's role [42].

### Communicating the diagnosis and ethical concerns

Communication of the diagnosis of dementia was addressed in eight of the CPGs [41, 42, 44, 46, 50, 51, 54, 55]. Three CPGs explicitly stated that the GP is the healthcare professional who should communicate the diagnosis [42, 50, 54], with two providing specific guidance on the process [50, 54]. Other CPGs were less specific; the Scottish SIGN CPG suggested the GP with multidisciplinary team involvement, while the Qatar CPG recommended a senior multidisciplinary team member. While not addressing the communication of the diagnosis overtly, the CPG from Germany suggested that the patient needs to be informed of a suspected diagnosis, offering the option of whether to proceed with a formal diagnosis [44].

Across the CPGs, several ethical challenges in dementia diagnosis were highlighted. These included balancing clear and sensitive communication [46], respecting patient autonomy and involving caregivers while maintaining confidentiality [44–46, 51], ensuring informed consent [42, 44, 51], managing potential psychological distress [41, 42, 50, 51, 54], assessing decision-making capacity [41, 48, 50] and navigating legal implications, such as driving [50, 54]. CPGs consistently encouraged healthcare professionals to provide ongoing support, consider individual patient needs, and approach these dilemmas with empathy.

### Referral

Referral criteria for suspected dementia varied considerably across the CPGs. A full summary of referral criteria is provided in Supplemental File S2. Three CPGs recommended referral when dementia is initially suspected [48, 49, 52], whereas others [41, 45, 46, 55] suggested that preliminary findings of the assessment should guide the decision whether to refer the patient. Two CPGs proposed delaying referral if dementia diagnostic criteria were not met, recommending reassessment after six months [42, 48]. The Scottish SIGN guideline specifically addressed the importance of the GP informing the patient of the reasons for a referral, in order to prepare them for the potential diagnosis of dementia [46].

Variability also existed regarding to whom the patient should be referred. The Netherlands CPG uniquely provided specific indications for referral to different specialists. Furthermore, CPGs from Ireland, the Netherlands, and the USA outlined what information the GP should include in a referral [50, 54, 55].

### Recommendations regarding anti-amyloid therapies and biomarkers

The use of AD biomarkers in the dementia process was mentioned across all CPGs, except for those specifically developed for primary care settings, from Ireland and the Netherlands [50, 54]. Biomarkers were considered important in the diagnosis of AD, with CSF analysis and PET imaging being the most discussed. Blood-based biomarkers were referenced in three CPGs from Italy, the USA, and Germany [41, 44, 55]. Although these CPGs did not explicitly recommend current use in clinical practice, they did acknowledge their future role in the diagnosis of dementia.

The potential future use of AATs for AD was acknowledged in the CPGs from Malaysia [48] and Latin America [45], identifying them as future treatment options that require further research. However, more recent CPGs from Italy [41] and the United States [55] provide a more comprehensive and up-to-date analysis of the implications of AATs. The Italian CPG highlights the potential modest benefits of these therapies while emphasising the need to consider serious risks, such as ARIA. The US CPG further discusses broader systemic challenges in the healthcare system, including the demand for additional expertise, resources, and multidisciplinary collaboration [55].

## Discussion

The global impact of dementia is increasing, positioning timely diagnosis as a critical public health priority. This scoping review systematically examined the role of GPs in the dementia diagnostic process as outlined in CPGs worldwide. This review highlights current recommendations, identifies variations in recommendations, and exposes important areas that require further clarification in CPGs. The findings underline the pivotal, yet frequently ambiguously defined, role of GPs as the first point of contact for individuals presenting with cognitive concerns.

While CPGs consistently position GPs as central to the identification of dementia, the guidelines themselves are predominantly designed for and by secondary care specialists, with minimal GP representation in their development. As our findings show, only two of the fifteen included CPGs were explicitly designed for a general practice audience [54, 56] and four had no reported GP involvement in their development [45, 47, 52, 53]. Notably, these documents (primarily consensus statements) also scored poorly on the “Rigour of Development” AGREE II domain, suggesting an association between a lack of GP stakeholder involvement and lower methodological rigour. Guideline development committees are often convened by specialist bodies, creating an inherent secondary-care perspective. Multidisciplinary guideline development, tailored to the practical realities of general practice, is essential for improving healthcare quality, consistency, and outcomes [57]. Furthermore, CPGs typically focus on a single disease, whereas the core of general practice is managing undifferentiated illness, multimorbidity, and psychosocial complexity [58]. A guideline that fails to account for a patient's co-morbidities is of limited utility to a GP. This exclusion leads to impractical guidelines with low applicability, which in turn may lead to low adoption rates in general practice [59]. This can be misinterpreted by specialist groups as a lack of engagement with the evidence base. Breaking this cycle is essential to creating guidance that is not only evidence-based but will also be adapted by GPs and implementable at the frontline of patient care.

While the reviewed CPGs consistently position GPs as central to the timely identification and initial assessment of dementia, they fail to provide the practical guidance needed for GPs to fulfil this role. CPGs recommend detailed clinical histories, cognitive testing, and physical examinations, yet almost universally ignore the most significant barrier in general practice: time. The standard 10–15 minute GP consultation is fundamentally incompatible with the comprehensive dementia work-up envisioned by these guidelines [60]. The reviewed CPGs largely failed to provide practical solutions to this challenge, other than a suggestion in the Norway CPG to delegate parts of the assessment to a broader team involving home visits and a suggestion of AHP involvement in the SIGN and Qatar Guidelines [42, 46, 51]. This omission renders many CPG recommendations aspirational rather than actionable. By failing to address the practical challenges of general practice, CPGs create unrealistic expectations, asking GPs to perform complex assessments without the necessary resources.

This review highlights considerable variability in the diagnostic approaches recommended within CPGs, particularly regarding investigations like blood tests and imaging. This variability is a reflection of the diverse national healthcare policies, resource availability, and the established roles of primary and secondary care in different countries [61]. This divergence becomes critically important when considering the global context of dementia, where over 60% of people with the condition live in Low- and Middle-Income Countries (LMICs) [62]. In many LMICs, healthcare systems face severe constraints, including a lack of specialists, limited access to imaging or biomarker testing, and different cultural understandings of cognitive decline [63]. Therefore, a CPG from a high-resource setting that recommends investigations unavailable to the majority of the world's clinicians is not just impractical; it also risks creating a standard of care that is unattainable, potentially widening global health inequities. A truly useful comprehensive guideline would not prescribe a single, rigid pathway but rather a range of recommendations that can be adapted to local resource levels, ensuring that best practice is defined by what is feasible and beneficial in a given geographical context.

While the advent of blood-based biomarkers and AATs for Alzheimer's is scientifically significant, current CPGs offer almost no practical guidance for GPs on integrating their use into the diagnostic pathway. Inevitably, GPs will face an increased workload. This will involve a greater demand for cognitive evaluations, providing complex counselling on test results, explaining the modest benefits versus significant risks of AATs, and managing the mismatch between patient expectations and clinical reality [64]. Reviews of healthcare system preparedness for AATs have concurred that successful integration into clinical practice is dependent upon the direct involvement of general practice [65] [66]. There is an urgent need to resource general practice and develop integrated care pathways that ensure timely diagnosis and treatment of Alzheimer's disease in the biologic era, enabling equitable access to AATs [67]. This oversight in practical guidance in CPGs suggests that the necessary frameworks are not yet in place, leaving the GPs ill-equipped to manage the profound changes that will accompany these innovations.

### Strengths and limitations

To our knowledge, this is the first review to examine the role of the GP in the diagnostic process within dementia CPGs internationally, providing critical insights and highlighting gaps and variations in recommendations. Our extensive search strategy, which included multiple peer-reviewed databases, grey literature sources, and professional organisations, ensured the retrieval of relevant and up-to-date CPGs from a diverse range of healthcare systems globally. We appraised the guidelines with careful consideration of the methodological differences between CPGs and consensus statements, ensuring the AGREE II domains used were of clinical relevance. However, a number of limitations must be acknowledged. While we translated non-English language CPGs using Google Translate, thereby increasing international representation, it is possible that some linguistic nuance may not have been fully captured. Although our search was limited to 5 years and updated before publication, the rapidly evolving pace of change in dementia diagnosis and care means that CPGs were potentially representative of different diagnostic eras. Finally, the applicability of findings may be influenced by differing healthcare systems, policies and resource availability internationally.

## Conclusions

This review highlights the urgent need for updated and standardised recommendations within CPGs, providing explicit, practice-relevant guidance for GPs. International GP organizations should collaborate to develop a primary-care-focused CPG for dementia that can be adapted to different national health systems. GPs should advocate for and adopt structured tools to improve the quality and efficiency of dementia assessment. This includes the use of standardized referral templates that ensure all necessary clinical information is conveyed to specialist services.

Updated recommendations should include clear criteria for the integration of biomarker and disease-modifying therapies and standardized, streamlined referral pathways to specialist memory clinics. Crucially, CPGs should also outline criteria for patients not requiring specialist referral or biomarker-supported diagnoses, ensuring that general practice can effectively care for this large cohort of patients. Future research must focus on developing and evaluating shared care models for the implementation of novel biomarkers and AATs, defining the specific roles and responsibilities of primary and secondary care to ensure safe, equitable, and sustainable integration into practice. Policymakers must recognize that preparing general practice for this new era of dementia diagnosis requires significant investment in workforce training, infrastructure, and the development of new care pathways.

By collectively addressing these critical priorities, we can empower general practice to deliver expert, high-quality dementia care, ultimately improving outcomes for people living with dementia and their families.

## Supplementary Material

cmaf103_Supplementary_Data

## Data Availability

Data that support the findings of this study are available from the corresponding author upon reasonable request.
