# Supplementary material for: The role of general practitioners in dementia diagnosis: a scoping review of clinical practice guidelines
Source: Fam Pract. 2026 Jan 22;43(1):cmaf103. doi: 10.1093/fampra/cmaf103 (PMC12823279; doi:10.1093/fampra/cmaf103)
Supplement: cmaf103_Supplementary_Data [file cmaf103_supplementary_data.zip › Supplementary Files 1 & 2.pdf]

Supplementary File 1: Search terms and inclusion and exclusion criteria

Search strategy: PubMed

((((Dementia[MeSH Terms]) OR (dementia*[Title/Abstract] OR Alzheimer*[Title/Abstract] OR "cognitive impairment"[Title/Abstract] OR "Cognitive decline"[Title/Abstract] OR "cognitive dysfunction"[Title/Abstract])) AND ((Diagnosis[MeSH Terms]) OR (Diagnos*[Title/Abstract] OR screen*[Title/Abstract] OR "best clinical practice"[Title/Abstract] OR "disease management"[Title/Abstract] OR "principles of car*"[Title/Abstract] OR "comprehensive car*"[Title/Abstract] OR Refer*[Title/Abstract])) AND ((Practice Guideline[MeSH Terms]) OR (Guid*[Title/Abstract] OR "best practice*"[Title/Abstract] OR procedure*[Title/Abstract] OR "Clinical Practice Guideline*"[Title/Abstract] OR recommend*[Title/Abstract]))) AND (2019:2024[pdat]))

Inclusion and exclusion criteria

Inclusion criteria

Papers from the past five years (2019-2024).
Guidelines for the management or diagnosis of Mild Cognitive Impairment (MCI), early Alzheimer's Disease and related dementias.
Evidence-based guidelines produced by national and international groups to guide clinical practice following formal procedures for practice guideline development.
Relevant to the diagnosis of dementia in primary care. Relevance determined by the inclusion of:
Screening to identify patients with MCI or Dementia
Clinical assessment
Diagnosis
Elimination of other possible causes of symptoms
Investigation of dementia
Recommendations for referral
Guidelines in any language.

Exclusion criteria

Guidelines for specific populations, e.g. guidelines for dementia in young adults or those with ID.
Guidelines not evidence-based or where the guideline development is not transparent.
Regional guidelines.
Research studies, academic journal articles such as those examining guideline adherence, compliance, development, or evaluation.
Editorials.
Conference proceedings and theses.
Study Protocols.
Guidelines specific to genetic testing, risk factors, dementia prevention, post-diagnosis care, palliative care, treatment or therapies for the management of dementia.
Guidelines specific for other health professionals, i.e., nurses, Allied Healthcare Professionals, or caregivers.
Guidelines for use in in-patient hospital settings.
National strategies or policies.

Summaries of CPGs

Supplementary File 2: Summary of referral information

Table 5: Summary of referral information in included CPGs

CPG (Country)	Reasons/criteria for referral	Referral made by	Referral to	Referral information
Argentina	• Time of evolution less than six months<br>• Rapidly progressive deterioration<br>• Patients under 65 years of age<br>• Atypical presentations<br>• Fluctuating or torpid evolution<br>• Positive signs on neurological examination<br>• Uncharacteristic findings in images<br>• Major psychiatric symptoms (s/DSMIV)<br>• Adverse drug events	GP	Neurologists, psychiatrists and geriatricians	
Canada	• Patients with a positive corroborative history<br>• Those with later life emergent and sustained NPS	N/R	A primary or specialty care memory clinic	
Scotland	• Specific criteria not given - the CPG suggests that cognitive tests may help to identify who should be referred to secondary care.	GP	Memory Clinic	
Malaysia	• All patients with suspected dementia and patients who are cognitively impaired, but the criteria for dementia are not met.	N/R (but all workup is the role of GP)	Geriatric psychiatrist/psychiatrist, geriatrician or neurologist	
Latin America	• Impairment is identified through cognitive or functional assessment	Primary care	Neurologist, geriatrician, or psychiatrist	
Brazil	• Degenerative or vascular dementia<br>• MCI is probably due to AD (amnestic MCI)<br>• (Rapidly progressive dementia) (On an urgent basis)<br>• When there are diagnostic doubts about the cause of dementia	Primary care physician	Neurology, geriatrics or psychiatry	
Ireland**	• Confirmation of the diagnosis<br>Exclusion of other pathologies<br>Subtyping of dementia<br>Tailoring treatments to the specific dementia subtype	GP	Old Age Psychiatry, Gerontology, Neurology or a dedicated Memory Clinic (dependent upon locally available resources)	Include the results of cognitive tests

Norway	• Insecure about the examination and/or it is difficult to make a diagnosis in particularly complicated cases<br>• When the person is younger<br>• When the person has a Sami cultural background or a minority background,<br>• Where language, level of education and/or culture are barriers to the investigation,<br>• Where the specialist health service has better expertise in the area<br>• When the person has a developmental disability	GP	Specialist health service	
Netherlands**	• Need for certainty about the diagnosis of dementia.<br>• Diagnostics for people who do not speak Dutch (or cannot take the RUDAS<br>• Need for a nosological diagnosis (diagnostics focused on the type of dementia).<br>• (Suspicion of) dementia at a young age (arbitrarily < 65-70 years).<br>• Indications of a special form of dementia other than Alzheimer's disease or vascular dementia<br>• Suspicion of a (possibly) treatable cause for the cognitive decline<br>• Additional somatic or psychological factors<br>• Crisis intervention with or without the need for court authorization or custody	GP	*Memory Clinic<br>Geriatric Specialist,<br>Mental health psychologist,<br>Geriatric Psychiatrist,<br>Geriatric Medicine Specialist	A link is provided to information that should be included in a referral letter.
Qatar	• Where dementia with no underlying cause is suspected<br>• Any patient with suspected cognitive impairment based on initial assessment and informant history, supported by the results of the Mini-Cog Assessment.<br>• Patients who fail to improve after an underlying cause is treated.<br>(Note: Based on the referral from Primary Care, the Memory Clinic Team will determine referral to the subspecialist clinics)	GP makes a referral to a memory clinic. Memory clinic makes referrals to subspecialists.	Memory Clinic	

Turkey	• Patients with suspected Alzheimer's disease, Mild Cognitive Impairment or Subjective Cognitive Impairment	Not reported	Higher-level centres or centres with specific dementia outpatient clinics	
Italy	• When reversible causes of cognitive decline (including delirium, depression, sensory impairment [such as sight or hearing loss] or cognitive impairment from medicines associated with increased anticholinergic burden) have been investigated and dementia is still suspected.<br>• If the person has suspected rapidly progressive dementia, refer them to a neurological service with access to tests (including cerebrospinal fluid examination) for Creutzfeldt-Jakob disease and similar conditions.	GP	(Centre for Cognitive Disorders and Dementias)	Not reported
USA	• A patient with atypical findings or in whom there is uncertainty about how to interpret the evaluation, or that is suspected of having an early-onset or rapidly progressive cognitive-behavioral condition<br>• The primary care clinician is unsure about the interpretation or implication of abnormalities on the neurologic exam<br>• For patients in whom the history and clinician's pre-test concern are incongruent with the patient's performance on a brief cognitive test, biopsychosocial and other factors should be considered and a referral to a neuropsychologist or other specialist should be considered	GP	Neuropsychological evaluation	The referring clinician should provide<br>1)the specific cognitive or behavioral domains of concern to the patient, care partner, and/or referring clinician.<br>2) information about the patient's education, work experience, and any physical, medical or emotional comorbidities that may affect the patient's ability to provide a history or perform tests

* The CPG gives guidance on which HCP to refer to for specific need

**CGP was developed for the general practice/primary care setting.

Note: Finland and Germany did not include referral criteria
